# Retraction: Downregulated brain and muscle aryl hydrocarbon receptor nuclear translocator-like protein-1 inhibits osteogenesis of BMSCs through p53 in type 2 diabetes mellitus

**DOI:** 10.1242/bio.058581

**Published:** 2022-06-28

**Authors:** Xiaofei Mao, Xiaoguang Li, Wei Hu, Siwei Hao, Yifang Yuan, Lian Guan, Bin Guo

This notice updates and replaces the Expression of Concern (doi:10.1242/bio.05525).

The journal is retracting *Biology Open* (2020) **9**, bio051482 (doi:10.1242/bio.051482) because of concerns with reliability of the data.

After issues were raised by the Editor-in-chief with duplicated blots in Fig. 2B and Fig. 4A, and duplicated cell images in Fig. 2C and Fig. 5C, the authors were asked to supply all their original data. None of the original full blots could be found for any of the published figures despite the paper being published so recently and no explanations were given for the duplicated panels. The authors re-analysed their stored samples and supplied replicate blots and analyses, insisting that the conclusions of the paper remain sound. Raw data for all graphs and other cell images were supplied.

The journal concluded that without the original data for all the published figures, we have no option but to retract this paper. This course of action follows the advice set out by COPE (Committee on Publication Ethics), of which Biology Open is a member.

All authors agree with this retraction.

